# The hidden crisis: double burden of malnutrition among refugee children in South Asia – a systematic review and meta-analysis from observational studies

**DOI:** 10.3389/fnut.2024.1480319

**Published:** 2025-02-10

**Authors:** Pooja Panchal, Mohd Usman, Tajung Longkumer, Reshma Susan Babu, Mahalaqua Nazil Khatib, Shariza Abdul Razak, Kavitha Menon

**Affiliations:** ^1^Nutrition and Dietetics Program, Symbiosis School of Culinary Arts and Nutritional Sciences (SSCANS), Symbiosis International (Deemed University), Pune, India; ^2^Department of Community Medicine, Symbiosis Medical College for Women, Symbiosis International (Deemed University), Pune, India; ^3^Datta Meghe Institute of Medical Sciences (Deemed University), Wardha, India; ^4^Dietetics Programme, School of Health Sciences, Kota Bharu, Kelantan, Malaysia

**Keywords:** refugee children, South Asia, malnutrition, undernutrition, obesity, overweight, nutritional status

## Abstract

**Background:**

Children living in refugee camps in South Asian countries suffer from undernutrition. However, the emerging prevalence of double burden of malnutrition could potentially cause a crisis in the healthcare of the refugee population. Double burden increases the risk for co-morbidities, poor functional health, and increased risk for premature death among these children. The study aims to assess the prevalence of malnutrition among refugee children in South Asia.

**Methods:**

This systematic review and meta-analysis followed the standard Preferred Reporting Items for Systematic Reviews and Meta-Analyses (PRISMA) 2020 guidelines using CoCoPop mnemonic approach. We searched JSTOR, Scopus, PubMed, Web of Science, and MEDLINE databases for studies on the prevalence of malnutrition in refugee children from 1984 to August 2024 with restricted English language. The screening of research articles was undertaken using COVIDENCE 2.0 software. The JBI checklist was used to assess the methodological quality of the included articles. The meta analysis was carried out using MedCalc 22.018 software. The gray literature was manually searched from the reputed organizations focusing on refugee children and was narratively analyzed for malnutrition statistics. Furthermore, the corroboration of primary research articles and gray literature was conducted for comprehensive understanding.

**Results:**

The review included 10 full-text research articles, all with cross-sectional study design and 11 gray literatures. The 10 studies covered a total of 4,274 participants with 3,536 urban and 738 rural refugee children aged between 0 and 19 years [refugee children (*n* = 8) and refugee adolescents (*n* = 2)]. The sample size of the included studies varied between 58 and 1,087 and mostly from Bangladesh. The prevalence of stunting ranged from 3.9–75.4% in the included studies [pooled prevalence: 31.8% (95% CI: 18.6–46.6%)]; wasting between 0.3–24.3% [pooled prevalence:10.1% (95% CI: 4.6–17.3)]; underweight between 4.4–65% [pooled prevalence: 19.1% (95% CI: 10.8–29.2%)]; and overweight between 3 and 24% [pooled prevalence: 6.5% (95% CI: 2.6–12.1%)]. Time trend analysis of the prevalence of malnutrition showed a decreasing trend in underweight, an increasing trend for stunting and wasting, and overweight including a rising prevalence of dual burden of under-and overnutrition.

**Discussion:**

The study indicates a high prevalence of undernutrition and a rising prevalence of overnutrition -the Asian paradox of the double burden of malnutrition in refugee children living in South Asia. The coexisting double burden of malnutrition among refugee children calls for comprehensive programs and policies for the prevention and management of the double burden of malnutrition.

## 1 Introduction

South Asia is hosting nearly 3.6 million refugees and asylum-seekers experiencing the “Asian paradox” of double burden of malnutrition (1). This paradox involves both high rates of undernutrition and an increasing prevalence of over nutrition, underscoring the urgent need for comprehensive strategies and policies to address these issues effectively. The United Nations High Commissioner for Refugees (UNHCR) defines a refugee as “an individual who has been compelled to flee their country due to persecution, war, or violence, and who has a well-founded fear of persecution based on race, religion, nationality, political opinion, or membership in a particular social group” (2). In the Asia-Pacific region, approximately 15.7 million displaced individuals—including refugees, asylum-seekers, stateless persons, and returnees—are under the UNHCR’s care, representing about 13 percent of the global refugee population (3). South Asia, in particular, hosts large numbers of refugees from Afghanistan and Myanmar, with Pakistan and Bangladesh being major host countries. Notably, children under 18 make up around 49% of the refugee population in this region (3). These children and their families typically reside in temporary camps or settlements, supported by the UNHCR, local governments, or various non-governmental organizations.

The process of migration can severely impact the health and nutrition of children, affecting their growth and development during critical years. Refugee children often suffer from chronic undernutrition in their home countries due to prolonged exposure to conflict and trauma. This situation is further exacerbated during their migration, increasing their susceptibility to infectious diseases, malnutrition, and higher mortality rates. In refugee camps, children commonly face food insecurity, inadequate clean water, poor shelter, and limited healthcare access, which worsens malnutrition. Additionally, adapting to new, resource-scarce environments and struggling to obtain sufficient and appropriate food can be particularly challenging and distressing (4). While previous studies from South Asia have highlighted high rates of undernutrition among refugees (5), recent research has also identified rising cases of overweight and obesity among these children (6, 7). This trend indicates a growing prevalence of the double burden of malnutrition—characterized by the coexistence of undernutrition (such as stunting, wasting, and underweight) and overnutrition (including overweight, obesity, and diet-related noncommunicable diseases) (8).

The coexistence of undernutrition (i.e., multiple micronutrient deficiencies, underweight and childhood stunting and wasting) and obesity/overweight with diet-related non-communicable diseases known as double burden of malnutrition (DBM) is common among children living in low- and middle-income countries (Popkin, Corvalan and Grummer-Strawn, 2020), and refugee children migrated to developed countries (9, 10). The phenomenon of coexisting under and overweight and obesity is less reported among refugee children of South Asian countries. However, if the prevalence of double burden of malnutrition surge above the thresholds of public health significance, could potentially result in grave health outcomes including early onset of non-communicable diseases and premature death of young adults (11).

Previous systematic reviews have primarily focused on various forms of undernutrition, such as stunting, among refugee children in South Asia (12, 13). There is a paucity of information on the double burden of malnutrition in refugee children living in settlements of South Asian countries. The evidence from the present review would be beneficial for policy makers, national and international organizations that care for refugee children. Information from the present review may initiate necessary deliberations among different stakeholders to develop programs and policies in the host countries to prevent and manage different forms of malnutrition among refugee children. Therefore, the present review aimed to summarize and derive pooled estimates of the different forms of undernutrition and overnutrition among refugee children in South Asia.

## 2 Methodology

The present review was prepared to assess the prevalence of childhood malnutrition in refugee children living in eight South Asian countries (Afghanistan, Bangladesh, Bhutan, India, Maldives, Nepal, Pakistan, Sri Lanka) (14). Additionally, the primary studies were corroborated with the existing gray literature published by reputed organizations [United Nations High Commissioner for Refugees (UNHCR), UNICEF, Centre for Disease Control and Prevention (CDC), Internal Displacement Monitoring Centre (iDMC), IMPACT, Plan International, and Action Against]. The approach adhered to the Preferred Reporting Items for Systematic Reviews and Meta-Analysis (PRISMA) 2020 guidelines for conducting systematic review (15).

### 2.1 Search strategy and selection criteria

All studies published in English between 1980 and 2024 were included in the review. A comprehensive search of original articles was performed using five electronic bibliometric datasets- JSTOR, SCOPUS, PubMed, Web of Science and MEDLINE. The following terms and keywords were used in the search: Refugee: “refugee’s”[All Fields] OR “refugees”[MeSH Terms] OR “refugees”[All Fields] OR “refugee”[All Fields] AND children: “child”[MeSH Terms] OR “child”[All Fields] OR “children”[All Fields] OR “child’s”[All Fields] OR “children’s”[All Fields] OR “children’”[All Fields] OR “child’”[All Fields] AND South Asia: “asia, southern”[MeSH Terms] OR (“asia”[All Fields] AND “southern”[All Fields]) OR “southern asia”[All Fields] OR (“south”[All Fields] AND “asia”[All Fields]) OR “south asia”[All Fields] OR “Afghanistan”[All Fields] OR “Bhutan”[All Fields] OR “Pakistan”[All Fields] OR “India”[All Fields] OR “Bangladesh”[All Fields] OR “Nepal”[All Fields] OR “Maldives”[All Fields] OR “Sri Lanka”[All Fields] OR “Maldives”[All Fields] AND Nutritional Status: “nutritional status”[MeSH Terms] OR (“nutritional”[All Fields] AND “status”[All Fields]) OR “nutritional status”[All Fields] OR “stunting”[All Fields] OR “wasting”[All fields] OR “underweight”[All Fields] OR “overweight”[All Fields] OR “obesity”[All Field].

### 2.2 Eligibility criteria

The inclusion criteria used the CoCoPop mnemonic (Condition, Context and Population) approach to determine the prevalence of nutritional status in refugee children of South Asian countries from the original articles (16).

The inclusion criteria for the peer-reviewed research articles included observational study designs (i.e., cohort, cross-sectional, and case–control designs) for the estimation of the pooled prevalence estimates. Exclusion criteria comprised of research articles focusing on the Internally Displaced persons (IDPs), returned IDPs, naturalized refugees, and resettled refugees. Using the above exclusion criteria gray reports solely defining the integrated care, published in language other than English, population greater than 19 years of age and lacking the inclusion of South Asian countries were not listed for the present review (Table 1).

**Table 1:** CoCoPop mnemonic approach.

Parameters	Inclusion	Exclusion
Context	South Asia (Afghanistan, Bangladesh, Bhutan, India, Maldives, Nepal, Pakistan, Sri Lanka) (14)	Non-South Asian countries
Condition	Prevalence of malnutrition based on anthropometric indicators underweight (WAZ < -2), stunting (HAZ < -2), wasting (WHZ < -2), and overweight (BMI-for-age Z score > +1 to +2 and WHA > 2) and -obesity (BMI-for-age Z Score > +2) (18)	Prevalence of malnutrition related to pre-existing conditions (any chronic or infectious diseases, genetic disorders, cognitive disorders, autoimmune disorders)
Population	Other people in need of international protection (OIP) refers to “*people who are outside their country or territory of origin, typically because they have been forcibly displaced across international borders, who have not been reported under other categories (asylum-seekers, refugees, people in refugee-like situations) but who likely need international protection, including protection against forced return, as well as access to basic services on a temporary or longer-term basis*” (39)	Children who live in normal zones and are unaffected by emergencies and Adults >19 years

### 2.3 Study identification and data extraction

The original research articles that were retrieved were uploaded to COVIDENCE 2.0, a systematic review software by Veritas Health Innovation (17) to conduct the review. This software facilitates an unbiased screening process for selecting research articles for inclusion in the systematic review. The selection of articles for current systematic review was completed in four steps: firstly, uploaded the RIS/PubMed files to the COVIDENCE 2.0 software and removed duplicates; secondly, two reviewers independently screened the title and abstracts (TL and RSB); thirdly, after retrieving the full-text article, duplicate screening of full-text articles was conducted based on inclusion eligibility criteria (TL and RSB); and finally, the included research articles were incorporated for the systematic review and meta-analysis. Disagreements regarding study inclusion for full-text review were resolved through discussion and consensus with a third co-author (KM) and discrepancies were resolved.

Two authors independently performed the data extraction from eligible full-text articles. Discrepancies in data extraction were addressed through a meeting at the end of double extraction, with unresolved issues settled by PP. A Microsoft Excel spreadsheet was used for data extraction, and tables were prepared. From the eligible studies, information was collected, including the author’s name, publication year, study location, country of residence for refugees, study design, number of participating refugee children, and prevalence of malnutrition in four categories: underweight, stunting, wasting, and overweight/obesity in refugee children up to 19 years of age. The gray literature data extraction included reports stating the title of report, agency of publication, age of population, prevalence data of underweight, overweight, stunting and wasting.

### 2.4 Critical appraisal checklist for studies reporting prevalence data

The Joanna Briggs Institute (JBI) critical appraisal tool for systematic reviews was used for critical appraisal of each article included in the study (16). The JBI critical appraisal tool for prevalence studies had nine questions in the checklist, and each question fell into one of four criteria: ‘yes,’ ‘no,’ ‘unclear,’ or ‘not applicable.’ Each checklist score was tallied and could have a maximum score of nine and a minimum of zero. A score between 0 and 3 was considered as low, 4–6 as medium, and 7–9 as high.

### 2.5 Data analysis

The data extracted was summarized based on the objective of the present review. The characteristics of the included studies were summarized, including author, year, age group of children, country of origin, sample size, and sub-categorized into rural–urban locations. Further, the prevalence of undernutrition underweight (WAZ < -2), stunting (HAZ < -2), wasting (WHZ < -2) and overnutrition indicators such as overweight (BMI-for-age Z score > +1 to +2 and WHA > 2) and obesity (BMI-for-age Z Score > +2), from the included studies were presented in the tables (18).

Meta-analysis was performed to estimate the pooled prevalence of malnutrition. A pooled estimate of underweight, stunting, wasting, and overweight prevalence was derived using a random-effects model (Dersimonian–Laird method) using MedCalc statistical software (version 22.018, MedCalc Software Ltd., Belgium). The random-effects model was used due to higher study variations. The MedCalc software was used for developing forest plots and analyzing the heterogeneity of the included studies (19). Given the diversity in geographical locations, sample sizes, participant ages, and outcome indicators across the retrieved studies, statistical heterogeneity was evaluated using Cochran’s Q and I^2^ statistics (I^2^ > 75% indicates substantial heterogeneity). Publication bias was evaluated using Egger’s test and Begg’s rank test and represented as the Funnel plot; a *p*-value <0.05 indicated the publication bias. Similarly, reports from various organizations supporting the welfare of the refugee children, migrants, and international agencies were listed manually. The presence of data from the gray literature was presented as “▲” (available) and “-” (unavailable). The data for gray literature was narratively synthesized.

## 3 Results

### 3.1 Data search and screening results

The study analyzed 10 research articles and 11 gray literature reports that met the inclusion criteria. The results were compiled and analyzed through meta-analysis for the prevalence of malnutrition, while gray literature was summarized narratively.

**Figure 1:**
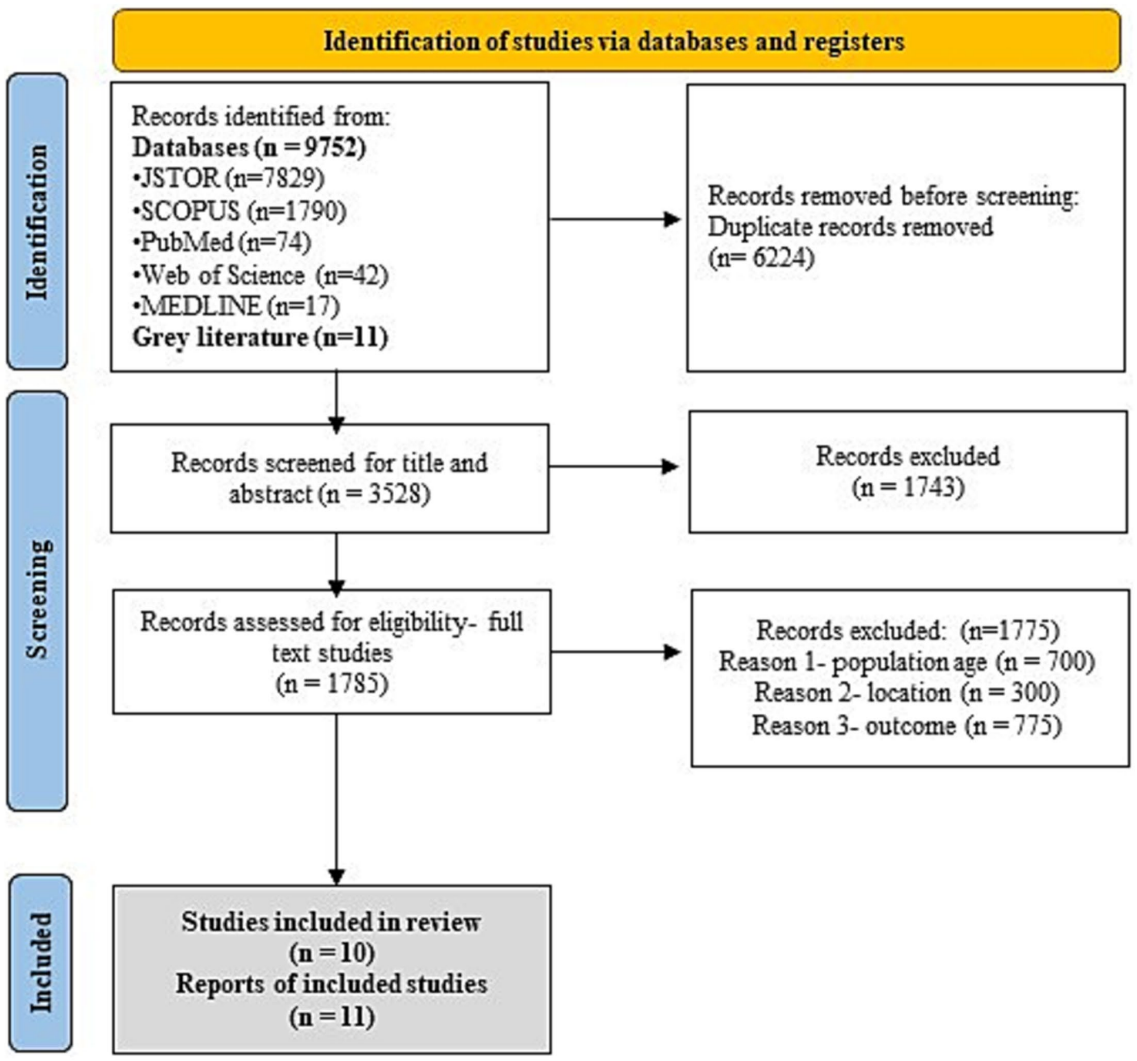
PRISMA flowchart.

A total of 9,752 research articles were retrieved from five databases from 1980 to 2024. The *Covidence 2.0* software was used for duplicate screening of research articles. Out of the 9,752 articles imported to the *Covidence 2.0* software, 6,224 were identified as duplicates. Subsequently, the title and abstract screening were conducted for the remaining 3,528 articles, resulting in 1743 exclusion as the primary research articles did not conform to the CoCoPop mnemonic approach. The remaining 1785 research articles were further screened for full text based on eligibility criteria. About 1775 articles, 700 articles for non-matching age groups of participants, 300 studies for non-South Asian locations (countries), and 775 research articles on not defining the prevalence values of under and over-nutrition were excluded. Finally, 10 full-text research articles, all with cross-sectional study design were included in the systematic review (6, 7, 20–27) (Figure 1). Additionally, gray literature consisting of reports from reputed organizations (*n* = 11) were included in the present review for strengthening the current review on the prevalence of malnutrition in refugee children from South Asian countries (28–37) (Figure 1).

### 3.2 Critical appraisal

**Figure 2:**
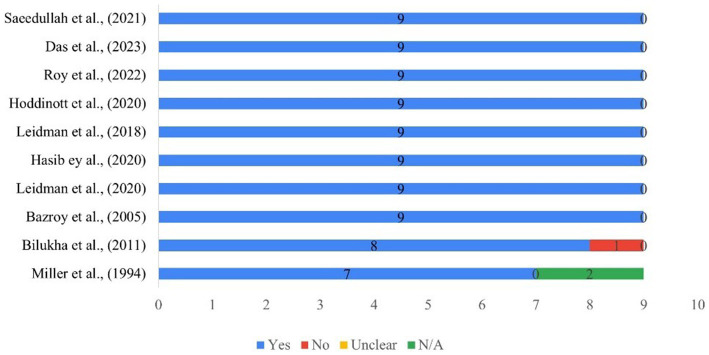
JBI scores for prevalence studies.

A quality assessment of the included primary research articles was conducted using the JBI checklist for prevalence studies and the results showed that most studies were of high quality (a score of >7 out of 9). Out of 10 included studies, eight studies had complied on all the nine critical appraisal questions (i.e., the studies included sample frame to address the target population, analysis of the samples, sample size, study settings, coverage of the data, identification of the conditions, standard and reliable methods for study participants, appropriate statistical analysis and response rates of the participants) (Figure 2).

### 3.3 Characteristics of the included studies

The included studies represented four countries in the South Asian region: Afghanistan (*n* = 2), Bhutan (*n* = 1), Bangladesh (*n* = 6) and India (*n* = 1) (Table 2). The 10 studies included in the present review covered a total of 4,274 participants with 3,536 urban and 738 rural refugee children, and the studies followed a cross-sectional study design. The ages of the participants ranged from 0 to 19 years [refugee children (*n* = 8) and refugee adolescents (*n* = 2)]. Of the 10 studies, eight were from urban and two from rural locations. The sample size of the included studies varied between 58 (from Afghanistan) to 1,087 (from Bangladesh) refugee children. Most of the studies covered childhood prevalence of malnutrition among refugee children (*n* = 6) from Bangladesh. All included studies were published between 1994 and 2023 (Table 2).

**Table 2:** Characteristics of refugee children living in South Asian countries.

No	Author (Year)	Country of origin	Study settings	Study design	Population	Age (Years)	Sample size
Urban							
1	Das et al. (6)	Bangladesh	Urban	Cross-sectional	Children, Adolescent	0–17	547
2	Roy et al. (27)	Bangladesh	Urban	Cross-sectional	Children	0.6–4.9	645
3	Hasib et al. (22)	Bangladesh	Urban	Cross-sectional	Children	0–5	100
4	Leidman et al. (25)	Bangladesh	Urban	Cross-sectional	Children	0.6–4.9	1,087
5	Leidman et al. (24)	Bangladesh	Urban	Cross-sectional	Children	0.6–4.9	269
6	Bilukha et al. (21)	Bhutan	Urban	Cross-sectional	Children	0.6–4.9	569
7	Bazroy et al. (20)	India	Urban	Cross-sectional	Children	0–5	261
8	Miller (26)	Afghanistan	Urban	Cross-sectional	Children	0–11	58
Rural							
9	Saeedullah et al. (7)	Afghanistan	Rural	Cross-sectional	Adolescent	10–19	206
10	Hoddinott et al. (23)	Bangladesh	Rural	Cross-sectional	Children	0–2	532

### 3.4 Overall prevalence of malnutrition (undernutrition and over-nutrition)

The included studies used standard anthropometric indicators to assess childhood nutritional status. Indicators of malnutrition used included underweight (WAZ < -2), stunting (HAZ < -2), wasting (WHZ < -2) and overweight (BMI-for-age Z Score > +1 to +2 and WHZ > +2) and obesity (BMI-for-age Z Score > +2).

Seven included studies showed the prevalence of underweight (6, 7, 20–22, 26, 27); nine studies reported stunting (6, 7, 20–27) and eight studies reported for wasting (6, 7, 21–25, 27). On the other hand, three studies reported the prevalence of overweight (6, 7, 22), and two studies obesity among refugee children (6, 7) (Table 3).

**Table 3:** Prevalence of undernutrition in refugee children living in South Asian countries.

No	Author (Year)	Country of origin	Study settings	Study design	Population	Age (Years)	Sample Size (*n*)	Z Scores	Prevalence of stunting *n* (%)	*Z* scores	Prevalence of underweight *n* (%)	*Z* scores	Prevalence of wasting *n* (%)
Urban													
1	Das et al. (6)	Bangladesh	Urban	Cross-sectional	Refugee Children	0–5	299	HAZ < −2	94 (31.6)	WAZ < −2	81 (27.4)	WHZ < −2	36 (12.1)
					Refugee Adolescents	11–17	248	HAZ < -2 to −3	72 (28.8)	BMI-for-age Z Score < −2 to −3	25 (10.0)	–	–
2	Roy et al. (27)	Bangladesh	Urban		Refugee Children	0.6–4.9	645	HAZ < -2	486 (75.4)	WAZ < −2	40 (6.2)	WHZ < −2	2 (0.3)
3	Hasib et al. (22)	Bangladesh	Urban	Cohort	Refugee Children	0–5	100	HAZ	41 (41.0)	WAZ	18 (18.0)	WHZ	13 (13.0)
4	Leidman et al. (25)	Bangladesh	Urban		Refugee Children	0.6–4.9	1,087	HAZ	211 (19.4)	–	–	WHZ < −3	34 (3.13)
5	Leidman et al. (24)	Bangladesh	Urban	Cross-sectional	Refugee Children	0.6–4.9	269	HAZ < −2	117 (43.4)	–	–	WHZ < −2	65 (24.3)
6	Bilukha et al. (21)	Bhutan	Urban	Cross-sectional	Refugee Children	0.6–4.9	569	HAZ < −2	133 (23.4)	WAZ < −2	119 (20.9)	WHZ < −2	46 (8.1)
7	Bazroy et al. (20)	India	Urban	Cross-sectional	Refugee Children	0–5	261	HAZ	5 (3.9)	WAZ	62 (47.3)	–	–
8	Miller (26)	Afghanistan	Urban	Cross-sectional	Refugee Children	0–11	58	–	–	WAZ	38 (65.0)	–	–
Rural													
9	Saeedullah et al. (7)	Afghanistan	WAZ	Cross-sectional	Refugee Adolescents	10–19	206	HAZ < −2	73 (35.3)	BMI-for-age Z Score < −2	6 (4.4)	–	33 (15.9)
10	Hoddinott et al. (23)	Bangladesh	Rural		Refugee Children	0–2	532	HAZ < −2	175 (33.4)	–	–	WHZ < −2	82 (15.8)

Underweight (Weight-for-Age Z-Scores <−2), Stunting (Height-for-Age Z-Scores <−2), Wasting (Weight-for-Height-Z-Scores <−2), BMI-Body Mass Index.

The highest prevalence of stunting and wasting was reported in in refugee children living in Bangladesh (75.4, 24.3%, respectively) (26, 27), and underweight (65%) in Afghani children (24). An Indian study reported the lowest prevalence of stunting (3.9%) (20). Wasting remained as the nutritional challenge - Bangladesh refugee children showed the highest prevalence of wasting (24.3%), followed by Afghanistan refugee children (15.9%) (7, 24). Among refugee adolescents, those from Afghanistan had the highest level of stunting (35.3%), followed by Nepal (26.9%) (Table 3).

Notably, half of the studies reported a very high prevalence of stunting of public health emergency (i.e.,>30%) (6, 7, 22–24, 27). Further, two studies reported high (20–30%) (6, 21), and one medium prevalence (10–20%) (25) of public health concern. Similarly, three out of eight studies reported a very high prevalence of wasting (>15%) (7, 23, 24), two studies high (10–15%) (6, 22), and one study medium (5–10%) (21) prevalence of public health emergency (Table 3).

Surprisingly, two studies from Bangladesh and one study from Afghanistan reported overweight and obesity among refugee children (6, 7, 22). Two authors studied refugee children from urban areas (6, 22), while one study focused on refugee children from rural areas (7). Afghan rural refugee adolescents had the highest prevalence of overweight (24%) and obesity (5.2%) than urban studies (7). Overnutrition was prevalent among refugee adolescents compared to refugee children under five (6, 7) (Table 4).

**Table 4:** Prevalence of overweight and obesity in asylum seeker children living in South Asian countries.

No	Author (Year)	Country of origin	Population	Age (Years)	Sample size (n)	Prevalence of overweight *n* (%)	Prevalence of obesity *n* (%)
Urban							
1	Das et al. (6)	Bangladesh	Adolescents girls	11–17	248	12 (5.0)	2 (0.7)
2	Hasib ey al. (2020)	Bangladesh	Children	0–5	100	3 (3.0)	-
Rural							
3	Saeedullah et al. (7)	Afghanistan	Adolescents	10–19	206	24 (24)	6 (5.2)

Overweight- BMI-for-age Z Score > +1 to +2 and WHZ >2, Obesity- BMI-for-age Z-Score > +2.

### 3.5 Estimates of pooled prevalence of stunting

**Figure 3:**
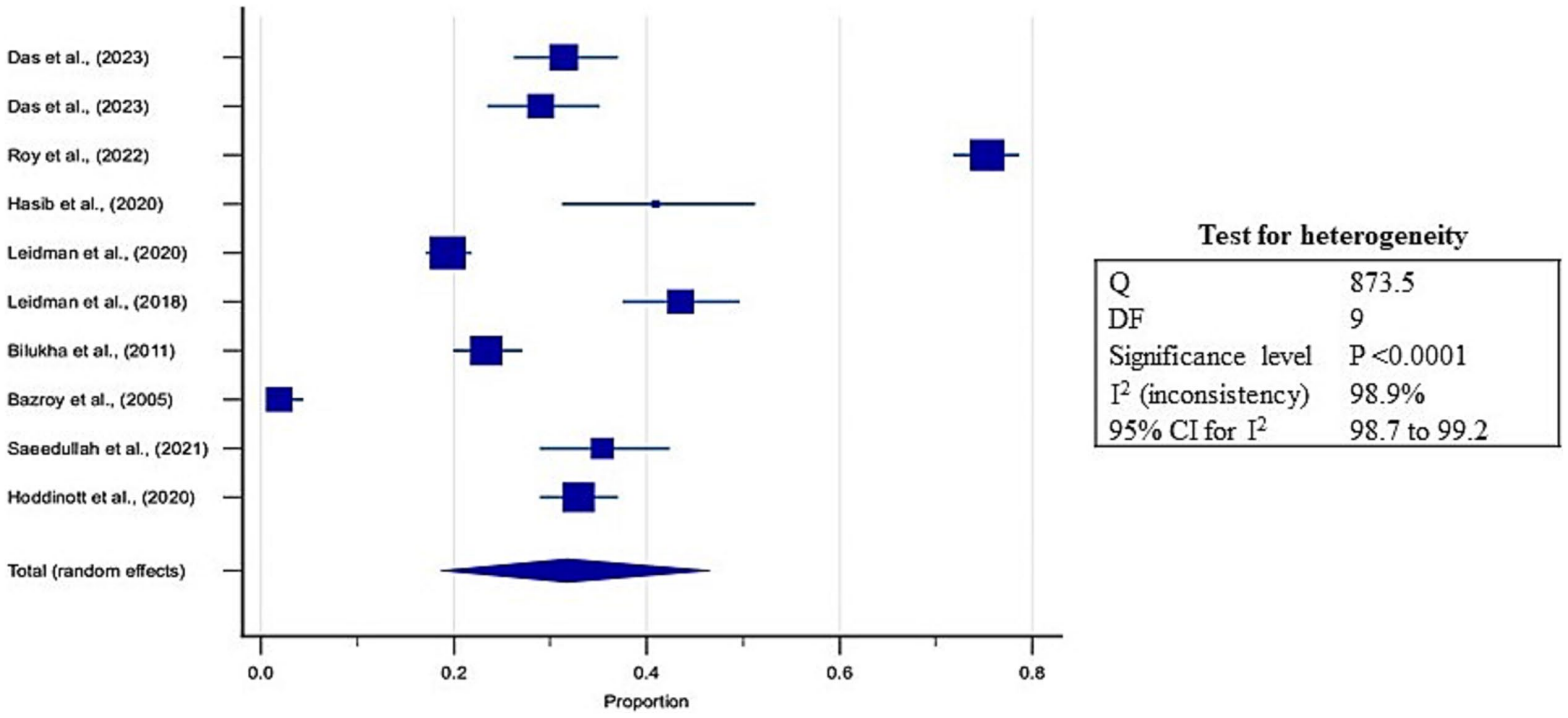
Forest plot for estimates of pooled prevalence of stunting in refugee children dwelling in South Asian Countries.

**Figure 4:**
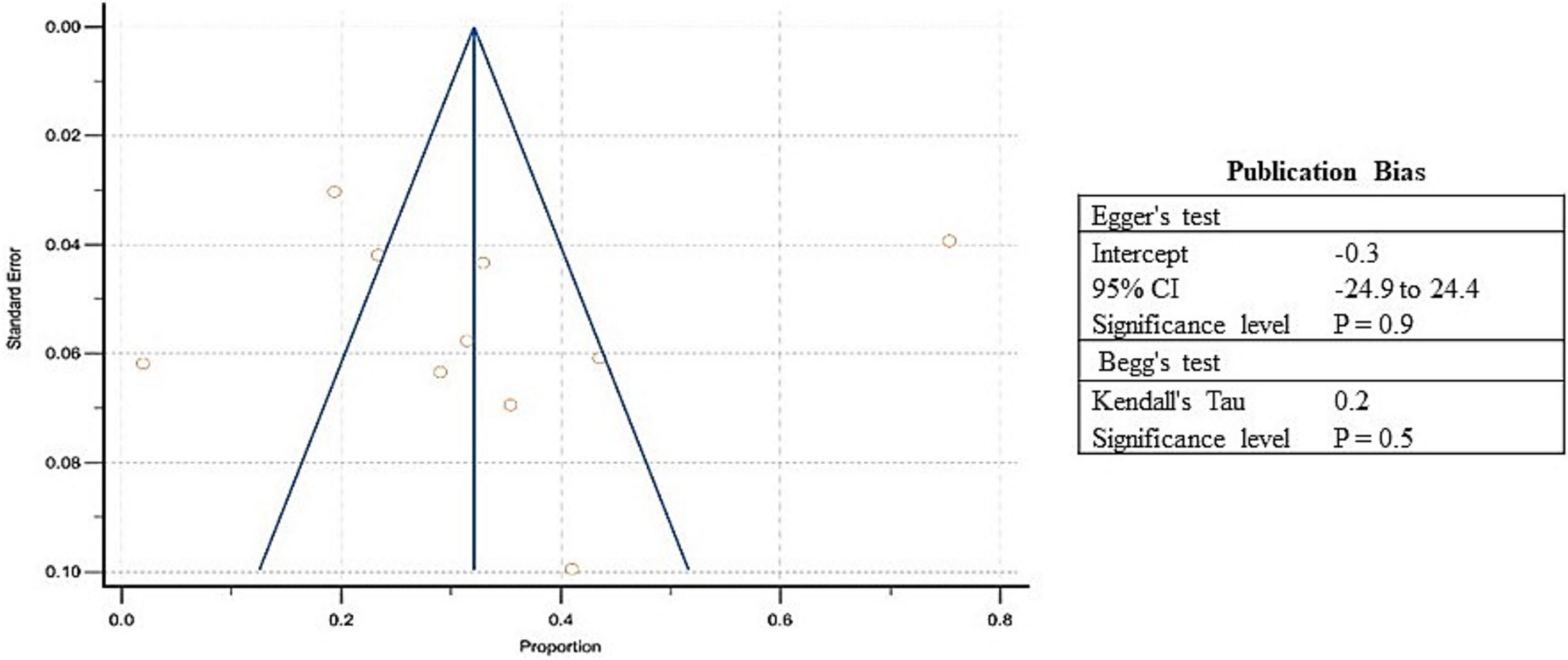
Funnel Plot showing publication bias for included studies of stunting among refugee children dwelling in South Asian countries.

Pooled estimates were calculated through meta-analysis software to summarize the prevalence of undernutrition and over nutrition in refugee children living in South Asia. Although the prevalence of stunting ranged from 3.9 to 75.4% in the included studies, the overall pooled prevalence of stunting was 31.8% (95% CI: 18.6 to 46.6%; Supplementary Table S1, Figure 3). A high heterogeneity was observed between the included studies (*I*^2^: 98.9); *p* < 0.0001 (Figure 3). The Egger’s test showed no statistically significant publication bias in the overall prevalence analysis of stunted refugee children (*p* = 0.9) (Figure 4).

### 3.6 Estimates of pooled prevalence of underweight

**Figure 5:**
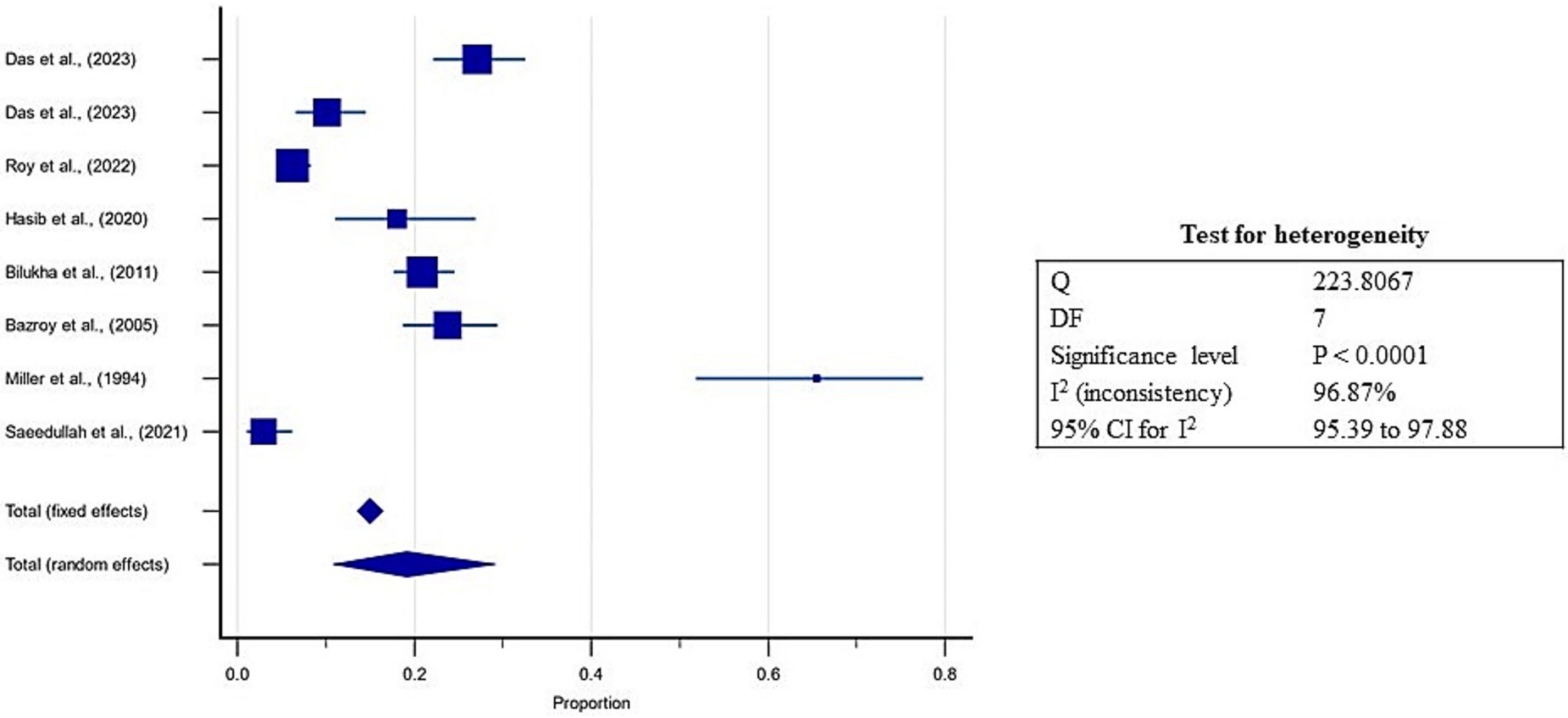
Forest plot for estimates of pooled prevalence of underweight in refugee children dwelling in South Asian Countries.

**Figure 6:**
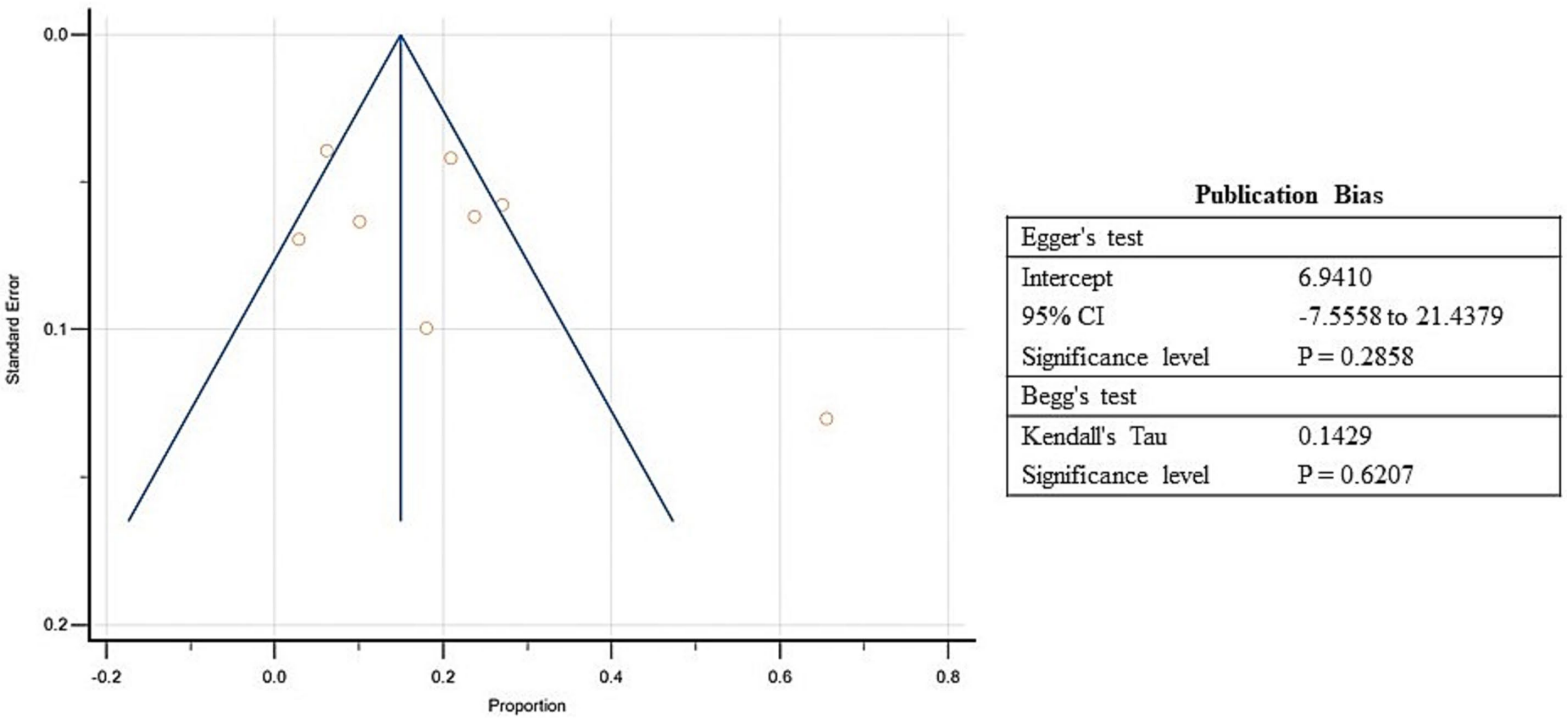
Funnel Plot showing publication bias for included studies of underweight among refugee children dwelling in South Asian countries.

Similarly, the prevalence of underweight varied between 4.4 to 65% in the included studies, and the estimated pooled prevalence of underweight was 19.1% (95% CI: 10.8 to 29.2%) (Supplementary Table S2; Figure 5). Higher heterogeneity was observed between the included studies (*I*^2^: 96.8%); *p* < 0.0001 (Figure 5). The Egger’s test showed no significant publication bias in the overall prevalence analysis for undernourished refugee children (*p* = 0.3) (Figure 6).

### 3.7 Estimates of pooled prevalence of wasting

**Figure 7:**
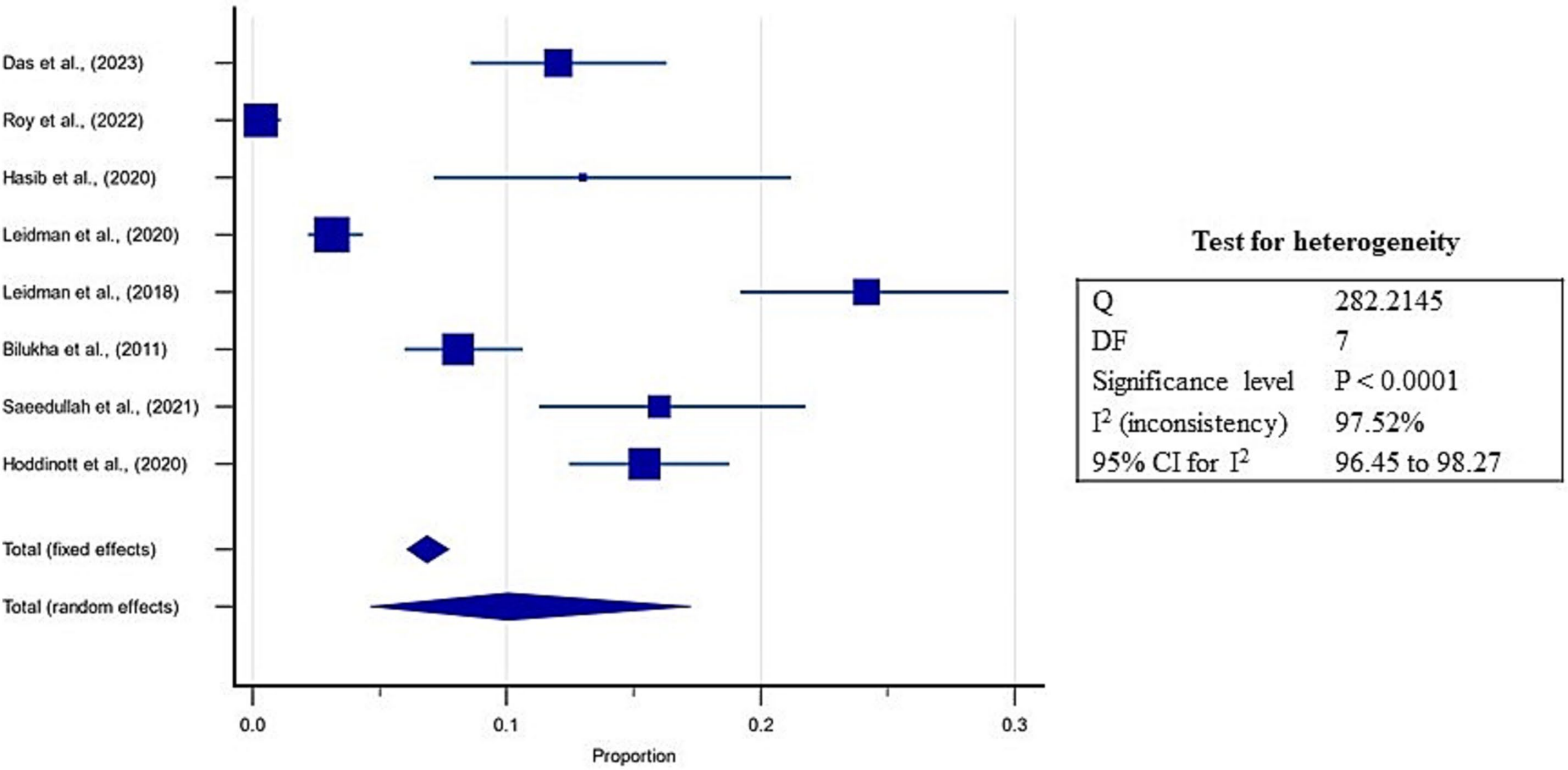
Forest plot for estimates of pooled prevalence of wasting in refugee children dwelling in South Asian Countries.

**Figure 8:**
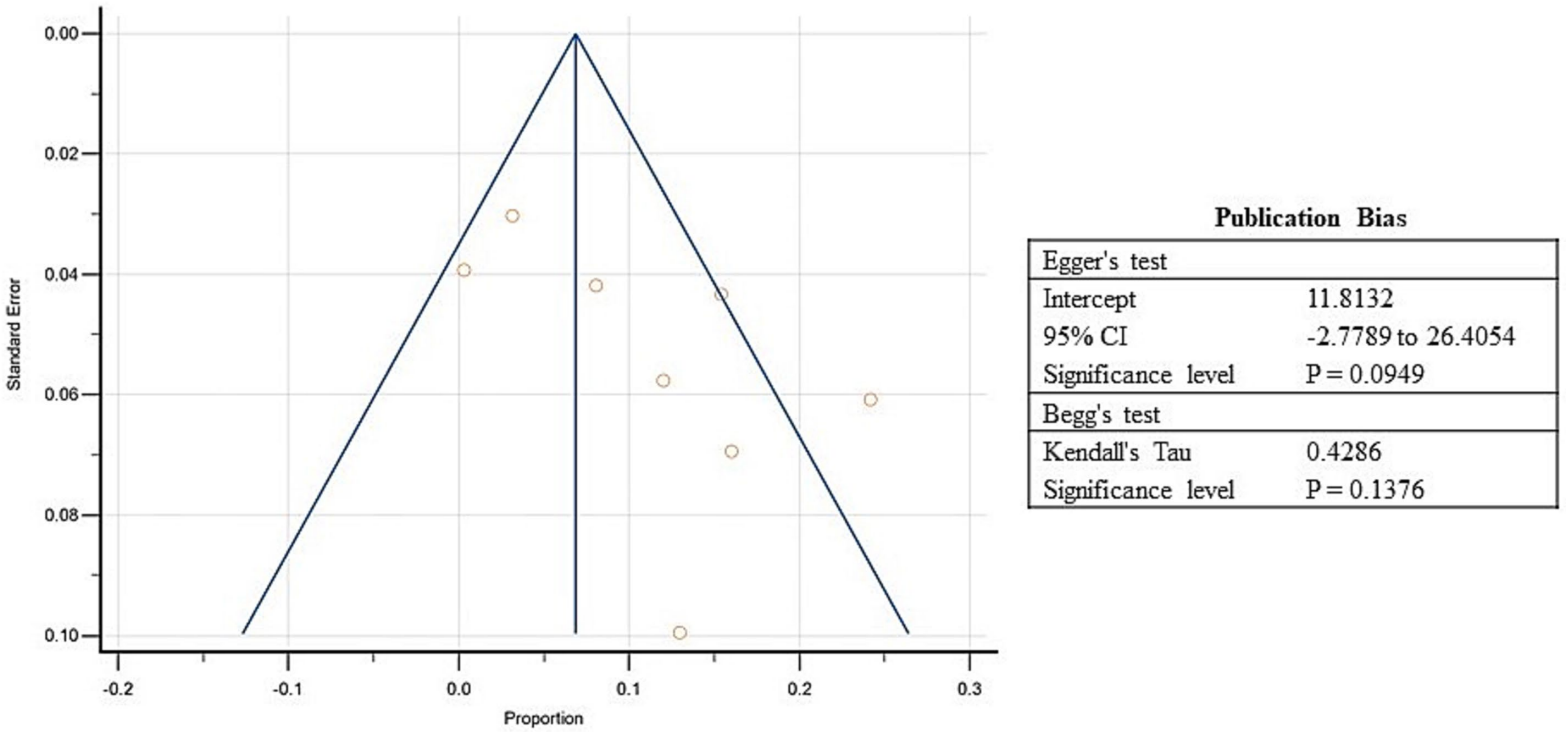
Funnel plot showing publication bias for included studies of wasting among refugee children dwelling in South Asian countries.

Further, the prevalence of wasting ranged from 0.3 to 24.3% in the included studies, and the estimated pooled prevalence of wasting was 10.1% (95% CI: 4.6 to 17.3%; Supplementary Table S3, Figure 7). A high heterogeneity was observed between the included studies (*I*^2^: 97.5%); *p* < 0.0001 (Figure 7). The Egger’s test showed no significant publication bias in the overall prevalence analysis for wasted refugee children (*p* = 0.09; Figure 8).

### 3.8 Estimates of pooled prevalence of overweight

**Figure 9:**
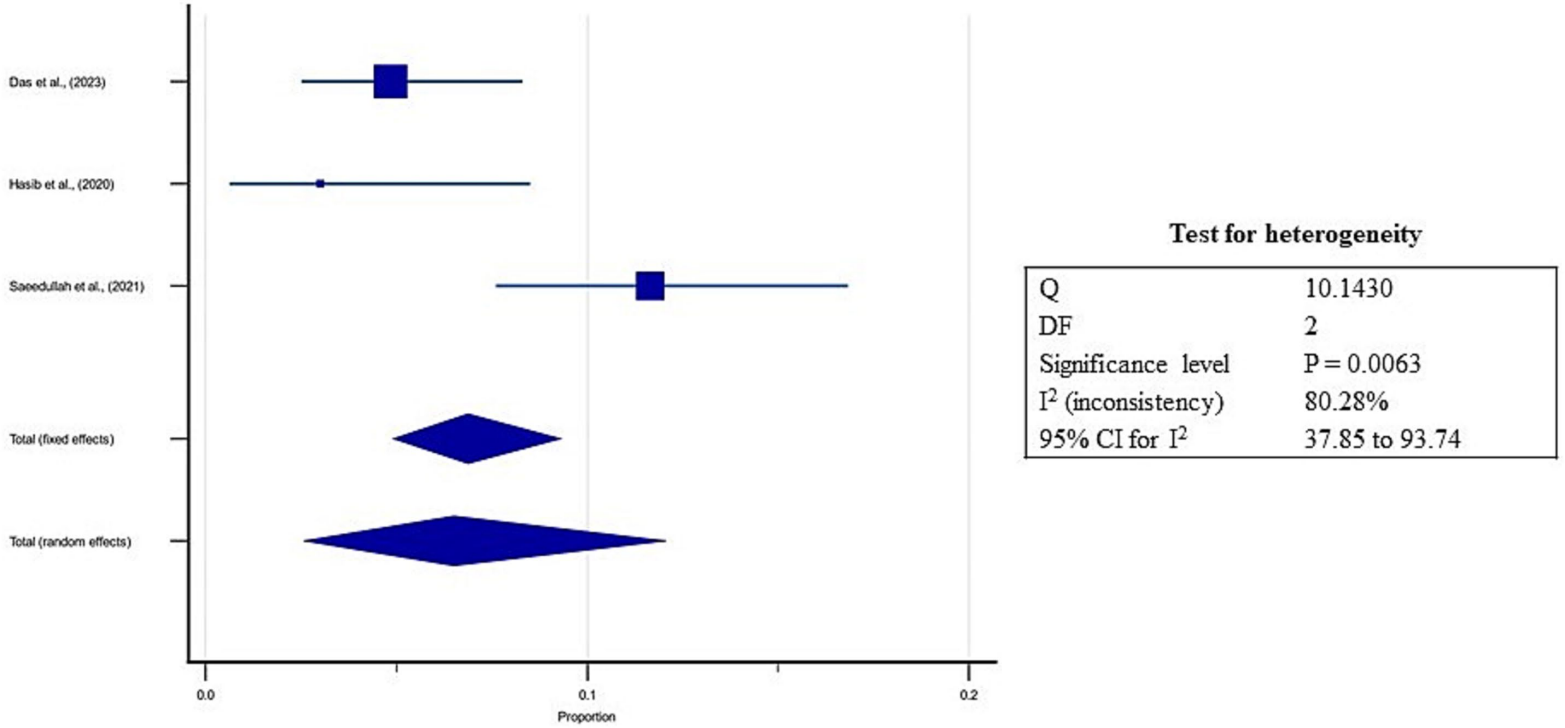
Forest plot for estimates of pooled prevalence of over nourishment in refugee children dwelling in South Asian Countries.

**Figure 10:**
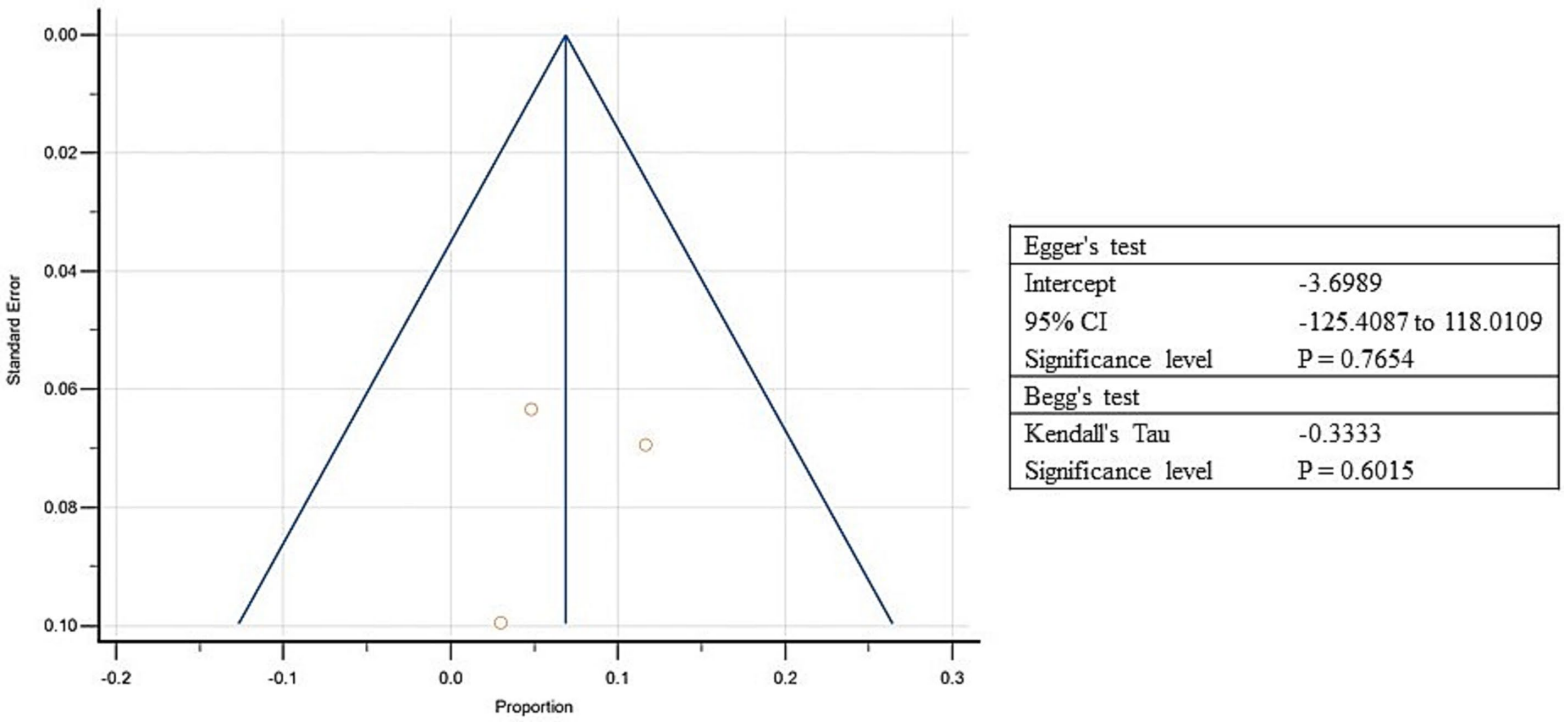
Funnel plot showing publication bias for included studies of over nourishment among refugee children dwelling in South Asian countries.

On the other hand, three studies from South Asia reported a prevalence of overweight between 3 to 24%, and the estimated pooled prevalence of overweight was 6.5% (95% CI: 2.6 to 12.1%; Supplementary Table S4, Figure 9). Higher heterogeneity was observed between the included studies (*I*^2^: 80.28%; *p* = 0.01; Figure 9). The Egger’s test showed no significant publication bias in the overall prevalence analysis for over nourished refugee children (*p* = 0.8; Figure 10).

### 3.9 Time series analysis of malnutrition in refugee children

Time trend analysis of the prevalence of malnutrition was conducted using the prevalence rates reported in primary research articles. The prevalence of underweight in refugee children reported a decreasing trend while that of stunting and wasting, showed an increasing trend. A limited number of studies reported the prevalence of overnutrition, yet an increasing trend of prevalence in refugee children was noted.

### 3.10 Gray literature

For the present review, gray literature was searched manually for information, in addition to the original research articles, on the prevalence of malnutrition among refugee children in South Asia. The gray literature included reports and policy documents from various organizations, and the search results were summarized. Eleven reports from international organizations were obtained after the screening - four were from the United Nations High Commissioner for Refugees (UNHCR), three from UNICEF, two from the Centre for Disease Control and Prevention (CDC), and one each from Internal Displacement Monitoring Centre iDMC, IMPACT, Plan International (31), and Action Against Hunger (28–37).

Most of the refugee children were found to be concentrated in Bangladesh and Pakistan. Around 500,000 Rohingya children sought refuge in camps in Bangladesh. Similarly, it was reported that 90% of refugees in South Asia were originally from Afghanistan (36). A majority of the literature reported on children under five years, and information on the nutritional status of refugee children between five and nineteen was underreported, or the availability of factual data was limited. UNICEF (35) reported a 7.5% prevalence of severe acute malnutrition among Rohingya refugee children, which was double the rate compared to their six-month prior reports (35). The opportunity to corroborate of the prevalence results from original studies with that from the gray literature was limited as the comparable data was unavailable.

Additionally, the Kutupalong and Nayapara camps of Bangladesh reported high wasting and stunting in the under-five children. Overall, the reports focused on the social, demographic, and other related challenges and implications of malnutrition in refugee children of South Asia. However, information (data) on the prevalence of malnutrition was limited in the gray literature that otherwise addressed the challenges of refugee children, including food insecurity (Table 5).

**Table 5:** Prevalence of overweight and obesity in refugee children living in South Asian countries.

No	Source (Year)	Title of report	Age (Years)	Under weight (%)	Over nutrition (%)	Stunting (%)	Wasting (%)
1	UNHCR/UN Women (42)	Afghanistan Crisis Update- Women and Girls in Displacement - Factsheet III	<5	–	–	–	–
2	UNICEF (36)	Children on the Move in South Asia Regional Brief	<5	▲	–	▲	–
3	iDMC, IMPACT, Plan International (31)	Women and girls in internal displacement	<5	–	–	–	–
4	(29)	Emergency nutrition assessment final report Nayapara and Kutupalong registered refugee camps and makeshift settlements. (Kutupalong)	<5	▲	–	▲	▲
		Emergency nutrition assessment final report Nayapara and Kutupalong registered refugee camps and makeshift settlements. Nayapara	<5	▲		▲	▲
5	UNICEF (35)	Malnutrition rates among Rohingya refugee children in Bangladesh appear to be at least double earlier estimates	<5	–	–	▲	–
6	UNHCR (33)	Solutions Strategy for Afghan Refugees	<5	–	–	–	–
7	UNHCR (33)	UNHCR Operational Guidance on the Use of Fortified Blended Foods in Blanket Supplementary Feeding Programs	<5	▲	–	▲	–
8	CDC (28)	Malnutrtion and Micronutrient Deficiencies Among Bhutanese Refugee Children- Nepal 2007	<5	▲	–	▲	▲
9	UNHCR/WHO (32)	The road to health and the road to Afghanistan	<5	▲	–	▲	▲
10	UNICEF (37)	Children affected by armed conflict in South Asia: A review of Trends and issues identified through secondary research	<5	–	–	–	–
11	CDC (30)	Nutritional Assessment of Adolescent Refugees- Nepal 1999	10–19	▲	–	–	–

CDC, Centre for Disease Control and Prevention; UNHCR, United Nations High Commissioner for Refugees; iDMC, Internal Displacement Monitoring Centre; UNICEF, United Nations International Children’s Emergency Fund.

## 4 Discussion

The present study proposed estimating malnutrition prevalence in refugee children living in South Asian countries. The silent findings of the present systematic review and meta-analysis were: (1) the studies reported predominantly from Bangladesh, especially post 2017 Myanmar crisis; (2) wide variations existed in the prevalence of malnutrition indicators and pooled prevalences are estimated; (3) most of the studies reported undernutrition than overnutrition status; and (4) the pooled estimates indicated double burden of malnutrition among the refugee children of South Asia. All the studies used valid, standard, and consistent measures of malnutrition in children, and studies were of high quality.

Evidence on the prevalence of malnutrition in refugee children predominantly was from Bangladesh. Although Pakistan hosts the highest number of refugees in South Asia (~19,88,231) than Bangladesh (9,71,984), most of the studies in the present review were from Bangladesh (3). Post 2017 refugee related publications in South Asia were predominantly from Bangladesh due to the Rohingya refugee crisis. Bangladeshi researchers have published several research articles on refugees, their food insecurity, nutritional status, and other diet-related challenges over the last 7 years, especially post the influx of about one million Rohingya refugees from Myanmar. Many researchers have probably had the opportunity to study refugees and report on the nutritional status of children to improve their lives and address their nutritional challenges. Also, children remain a vulnerable, dependent group- more than 60% of the refugees- and are most affected during their critical phase of growth and development (33).

The available evidence indicated wide variations in the prevalence rates of reported indicators such as stunting, underweight, and wasting, which made it necessary to have pooled estimates for the prevalence of malnutrition. The studies showed a higher prevalence of stunting than wasting (33.3% vs. 11.1%) among refugee children in South Asian countries. The present findings are identical to those of Skinner et al. (13), who evaluated the undernutrition among children under five living in refugee camps. According to the authors, severe wasting remains a significant problem in camps that needs urgent attention; often, persistent chronic malnutrition (stunting) is not well-addressed. Additionally, the stunting present in the children before they arrive in the refugee camps may not be amenable to the interventions offered in the camps (13).

Etiology of malnutrition in refugee children is multifactorial. A high rate of malnutrition is primarily attributed to the dependency of refugees on the humanitarian assistance with limited source or no livelihood (38). Additionally, poor access to good nutrition, the exposure to weather-related hazards such as flooding, cyclones, extreme heat and landslides; and unhygienic and congested living expose children to infectious disease that leads to the vicious cycle of malnutrition. Although the international agencies support the children with nutrition services for prevention and treatment of malnutrition, the impact remains modest in the crisis scenario (39).

Also, most of the studies in the present review reported undernutrition than overnutrition status, as most refugee camps have food insecurity and challenges of inadequate nutrition. However, two studies in the present review reported overweight and obesity among adolescent refugees in South Asia. The present study reported a pooled prevalence of 6.5% overweight. Similar results were reported among adolescents from refugee camps in high-income countries such as Korea (40) or upper-middle-income Turkey (9). Although the scenario is uncommon in South Asian countries, the high carbohydrate-rich diets with less diversified diets may have contributed to overweight and obesity among adolescents (6). Certainly, the rising trend needs attention to reduce the risk of non-communicable diseases.

The pooled estimates indicated a double burden of malnutrition among the refugee children of South Asia. Co-existing under and overnutrition indicate the need for programs to prevent and provide health care for nutrition-related comorbidities in the future. In their systematic review, Ankomah et al. (41) found a high prevalence of double the burden of malnutrition among migrants and refugees in developed countries (41). The effect was attributed to the double burden due to acculturation and the prevalence of unhealthy eating behaviors (4). The shift from a limited food environment for consumption in the home country to camps with near-adequate amounts of food in the host country improves the food intake. Further, physical inactivity and increased consumption of unhealthy or less diversified diets may lead to a double burden of malnutrition in refugee children (8).

There are a few strengths and limitations to the present work. The strengths include the inclusion of both research articles and gray literature on malnutrition of refugee children to provide a comprehensive overview of the scenario of South Asia. Also, the present study reported on the double burden of malnutrition in refugee children of South Asia – both undernutrition and overnutrition. The limitations include the risk of publication bias due to the inclusion of only articles published in English. However, a comprehensive search strategy and search of most of the electronic databases were conducted to capture most of the literature.

## 5 Conclusion

Most studies that evaluated the nutritional status of refugee or refugee children from Asian or developing countries reported severe undernutrition, as expected of the scenario in many settings. However, the present review reports the pooled prevalence of double burden of malnutrition in refugee children of South Asia. Further studies are needed to elucidate the potential predictors of the double burden of malnutrition and regular appropriate nutritional screening and focused nutrition education programs considering the potential implications in their adulthood.

## Data Availability

The original contributions presented in the study are included in the article/Supplementary material, further inquiries can be directed to the corresponding author/s.

## Supplementary material

SUPPLEMENTARY FIGURE S1 **(A-D)** Time trend analysis of stunting, underweight, wasting, and overweight in refugee children dwelling in South Asian countries.
