# Supplementary figures and images for: The hidden crisis: double burden of malnutrition among refugee children in South Asia – a systematic review and meta-analysis from observational studies
Source: Front Nutr. 2025 Feb 10;11:1480319. doi: 10.3389/fnut.2024.1480319 (PMC11847640; doi:10.3389/fnut.2024.1480319)

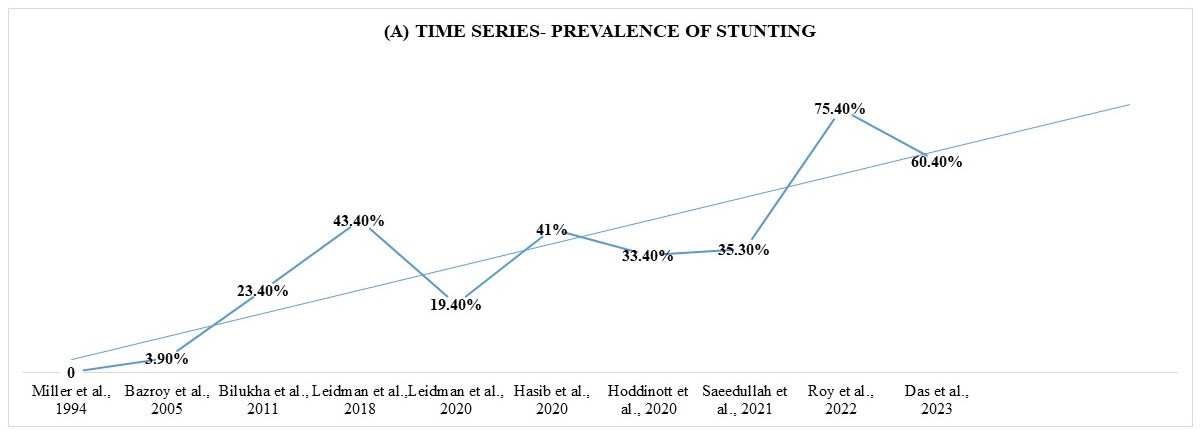

Supplement: SUPPLEMENTARY FIGURE S1 — (A-D) Time trend analysis of stunting, underweight, wasting, and overweight in refugee children dwelling in South Asian countries. [file Image_1.JPEG]

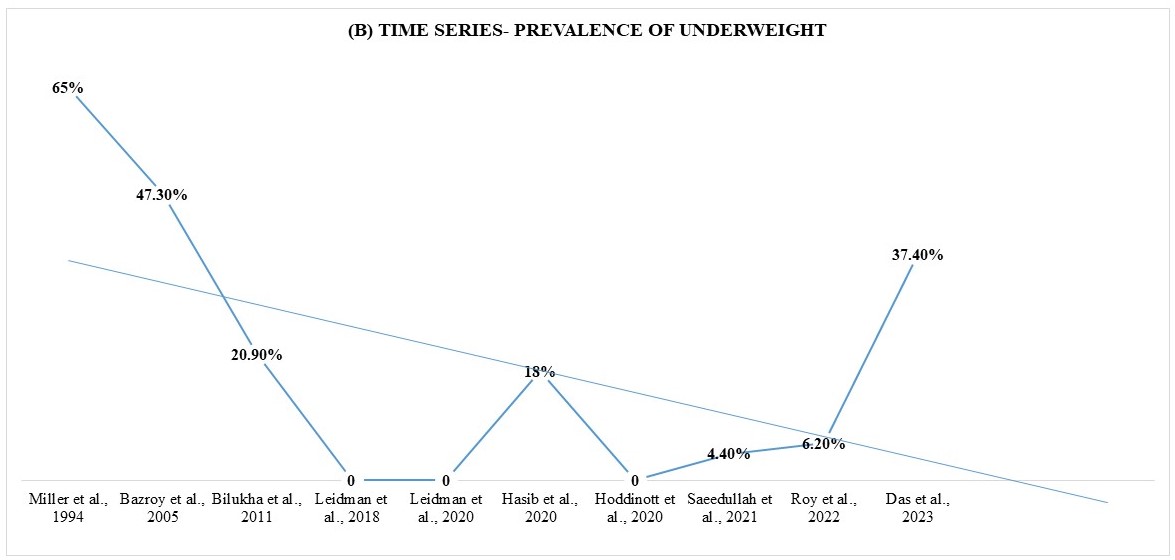

Supplement: Supplementary file 2 [file Image_2.JPEG]

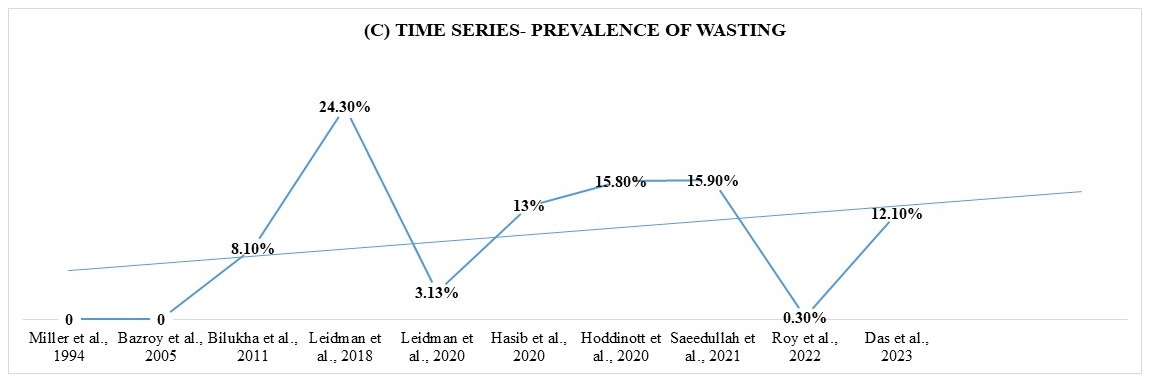

Supplement: Supplementary file 3 [file Image_3.JPEG]

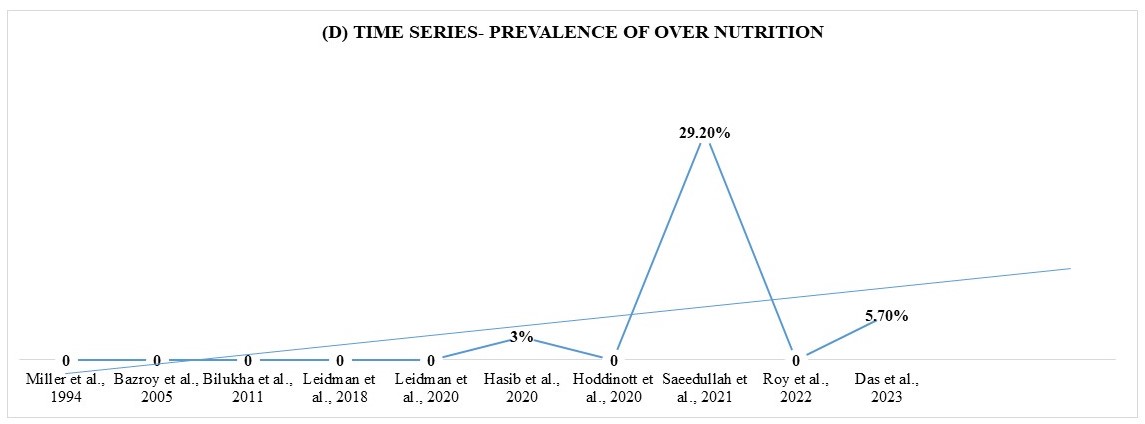

Supplement: Supplementary file 4 [file Image_4.JPEG]
