# Supplementary material for: The hidden crisis: double burden of malnutrition among refugee children in South Asia – a systematic review and meta-analysis from observational studies
Source: Front Nutr. 2025 Feb 10;11:1480319. doi: 10.3389/fnut.2024.1480319 (PMC11847640; doi:10.3389/fnut.2024.1480319)
Supplement: Supplementary file 5 [file Table_1.pdf]

Supplementary list of tables

Table 1. Meta-analysis: proportion of stunting in refugee children of South Asian countries

Study	Sample size	Proportion (%)	95% CI	Weight (%) Random
Das et al., (2023)	299	31.4	26.2 to 37.0	10.0
Das et al., (2023)	248	29.0	23.5 to 35.1	9.9
Roy et al., (2022)	645	75.3	71.8 to 78.6	10.1
Hasib et al., (2020)	100	41.0	31.3 to 51.3	9.7
Leidman et al., (2020)	1087	19.4	17.1 to 21.9	10.1
Leidman et al., (2018)	269	43.5	37.5 to 49.6	9.9
Bilukha et al., (2011)	569	23.4	19.9 to 27.1	10.1
Bazroy et al., (2005)	261	1.9	0.6 to 4.4	9.9
Saeedullah et al., (2021)	206	35.4	28.9 to 42.4	9.9
Hoddinott et al., (2020)	532	32.9	28.9 to 37.1	10.1
Total (random effects)	4216	31.8	18.6 to 46.6	100.0

Table 2. Meta-analysis: proportion underweight in refugee children of South Asian countries

**Study**	**Sample size**	**Proportion (%)**	**95% CI**	**Weight (%)** Random
Das et al., (2023)	299	27.1	22.1 to 32.5	12.7
Das et al., (2023)	248	10.1	6.6 to 14.5	12.7
Roy et al., (2022)	645	6.2	4.5 to 8.3	12.9
Hasib et al., (2020)	100	18.0	11.0 to 26.9	12.1
Bilukha et al., (2011)	569	20.9	17.6 to 24.5	12.9
Bazroy et al., (2005)	261	23.8	18.7 to 29.4	12.7
Miller et al., (1994)	58	65.5	51.9 to 77.5	11.4
Saeedullah et al., (2021)	206	2.9	1.1 to 6.2	12.6
Total (random effects)	2386	19.1	10.8 to 29.2	100.0

Table 3. Meta-analysis: proportion wasting in refugee children of South Asian countries

**Study**	**Sample size**	**Proportion (%)**	**95% CI**	**Weight (%) Random**
Das et al., (2023)	299	12.040	8.577 to 16.277	12.51
Roy et al., (2022)	645	0.310	0.0376 to 1.116	12.75
Hasib et al., (2020)	100	13.000	7.107 to 21.204	11.69
Leidman et al., (2020)	1087	3.128	2.176 to 4.344	12.84
Leidman et al., (2018)	269	24.164	19.172 to 29.734	12.46
Bilukha et al., (2011)	569	8.084	5.979 to 10.636	12.73
Saeedullah et al., (2021)	206	16.019	11.291 to 21.755	12.31
Hoddinott et al., (2020)	532	15.414	12.450 to 18.767	12.71
Total (random effects)	3707	10.050	4.625 to 17.258	100.00

Table 4. Meta-analysis: proportion overweight in refugee children of South Asian countries

Study	Sample size	Proportion (%)	95% CI	Weight (%) Random
Das et al., (2023)	248	4.839	2.525 to 8.300	35.80
Hasib et al., (2020)	100	3.000	0.623 to 8.518	29.44
Saeedullah et al., (2021)	206	11.650	7.609 to 16.838	34.76
Total (random effects)	554	6.507	2.574 to 12.065	100.00
